# TCMRD - KG: innovative design and development of rheumatology knowledge graph in ancient Chinese literature assisted by large language models

**DOI:** 10.3389/fphar.2025.1535596

**Published:** 2025-02-19

**Authors:** Haotian Li, Congmin Xia, Youjuan Hou, Sile Hu, Yanjun Liu, Quan Jiang

**Affiliations:** ^1^ School of Traditional Chinese Medicine, Beijing University of Chinese Medicine, Beijing, China; ^2^ Guang’anmen Hospital, China Academy of Chinese Medical Sciences, Beijing, China; ^3^ Institute of Information on Traditional Chinese Medicine, China Academy of Chinese Medical Sciences, Beijing, China; ^4^ Institute of Basic Theory for Chinese Medicine, China Academy of Chinese Medical Sciences, Beijing, China

**Keywords:** LLMS, trad. Chinese medicine (TCM), rheumatic, knowledge graph (KG), artifcial intelligence, data managament

## Abstract

**Introduction:**

Rheumatic immune diseases are a type of immune-inflammatory disease that affects muscles, bones, joints, and surrounding soft tissues. They have a long course and a high disability rate, seriously affecting the quality of life of patients. Traditional Chinese medicine plays an important role in the diagnosis and treatment of rheumatic immune diseases. The unique theoretical system and rich treatment methods of traditional Chinese medicine are preserved in ancient Chinese medical books.

**Methods:**

This study takes the content related to rheumatism in ancient traditional Chinese medicine books as the research object, integrates ontology theory and technology into the knowledge graph, and realizes the reconstruction of traditional Chinese medicine information knowledge. It provides a basic data structure for data mining and knowledge discovery.

**Results:**

This study is the first rheumatism-specific knowledge graph constructed based on ancient traditional Chinese medicine books. It has explored the construction method of a knowledge graph from ancient books by combining automatic labeling of mainstream large language models with manual review. Considering the knowledge characteristics of ancient traditional Chinese medicine books, where existing word segmentation technology struggles to accurately reproduce the original meaning, a new type of entity extraction method is proposed.

**Discussion:**

This provides an important foundation for improving the clinical diagnosis and treatment level of traditional Chinese medicine in treating rheumatism, further exploring the knowledge representation and application of traditional Chinese medicine in rheumatism treatment, and it has potential for future expansion and improvement.

## 1 Introduction

Rheumatism is a type of immune inflammatory disease that affects muscles, bones, joints, and surrounding soft tissues due to an impaired immune system (ALAMANOS et al., 2006). The global prevalence of rheumatism is over 6% (FENG et al., 2018), and more than 200 different types of rheumatism have been discovered. Its long course of disease and high disability rate significantly impact patients’ quality of life, imposing a substantial social and economic burden (Vestergaard et al., 2024). Traditional Chinese medicine (TCM) represents the unique wisdom of the Chinese people in life, health, and medical care, especially in the diagnosis and treatment of rheumatism, with a long history, unique theoretical system, and rich treatment methods (Jiang, 2023). With the development of computer technology and related theories, it is necessary to reorganize and utilize the knowledge and information of TCM in treating rheumatism through advanced modern technology, providing a basic data structure for data mining and knowledge discovery in the field of TCM.

A knowledge graph is a knowledge management model that emerged in the era of big data, which can fully leverage the importance of internal attributes and external connections of knowledge, helping to effectively integrate and mine TCM knowledge, and providing rich data for TCM research and clinical practice. Currently, some scholars in the field of TCM have constructed knowledge graphs in areas such as functional gastrointestinal diseases, health preservation in TCM, and prescription detection of Chinese patent medicines using natural language processing and machine learning technologies (Wang et al., 2020; Wang et al., 2021; Hongtao et al., 2021). However, the complexity, diversity, and ambiguity of TCM knowledge have increased the difficulty of accurately representing and correlating various types of knowledge, especially in the construction of knowledge graphs for ancient TCM texts, posing challenges to the modernization and intelligent application of TCM and hindering the effective utilization of related knowledge and information.

Ancient books of TCM refer to those medical books written or printed on paper carriers before 1911 (including 1911), containing a wealth of TCM theoretical knowledge and practical experience, and holding important academic and clinical value (Yang et al., 2023). Traditional methods of acquiring knowledge from ancient books rely on experts’ reading and research, which can no longer meet the current needs of TCM clinical and scientific research (Liu et al., 2024a). With the development of science and technology, the digital and networked organization of ancient TCM books using modern technology is the trend of modern development in TCM. In the process of constructing knowledge graphs for ancient TCM books, challenges still exist in accurately identifying entities, extracting relationships, and achieving comprehensive coverage of domain knowledge, with long-standing issues such as the loss of original meanings in ancient texts, weak associations between knowledge nodes, and insufficient granularity in entity types.

This study focuses on the content related to rheumatism in ancient TCM books, supplemented by classic TCM texts and related herbal formulas. It fragmented and categorized TCM classic knowledge through medical entity classification and knowledge element labeling and reconstructed the logical relationships between fragmented knowledge to form a knowledge graph. The work was led by the Institute of Basic Theory of Traditional Chinese Medicine, China Academy of Chinese Medical Sciences, in collaboration with Guang’anmen Hospital, China Academy of Chinese Medical Sciences, China Academy of Chinese Medical Sciences Institute of Information, Beijing University of Chinese Medicine, Liaoning University of Traditional Chinese Medicine, Yunnan University of Traditional Chinese Medicine, and Shenyang Aerospace University. The full content can be viewed at http://tcmrd-kg.cn/. The use of knowledge graphs can quickly find ancient literature and empirical knowledge related to the real world, and has already served TCM research, providing data support for TCM theory and clinical research.

## 2 Methods

### 2.1 Information extraction and data processing

#### 2.1.1 Scope of literature

This study included a systematic study of the Rheumatic immune diseases related contents of more than 378 ancient Chinese medical texts, which were categorized according to the categories in the General Catalogue of Chinese Ancient Medical Texts and were divided into twelve categories: “medical scriptures, basic theories, typhoid fever and the golden chamber, diagnostic methods, acupuncture, moxibustion and acupoint therapy, materia medica, formulae, clinical evidence of various disciplines, health maintenance, medical cases, medical treatments, medical treatments, medical histories, and comprehensive writings”. When organizing the literature, it covers the contents of “basic theory, etiology and pathogenesis, disease evidence, identification and treatment, therapy, diagnosis, acupuncture and moxibustion, acupoint therapy, medical cases, medical treatments and prescriptions, traditional Chinese medicine”, etc., and it will become an album of the collation of ancient literature on rheumatism (with a total of nearly 500,000 words). Additionally, the database included several classic works of traditional Chinese medicine, such as “Shang Han Lun” (Treatise on Cold Damage Disorders), “Ben Cao Gang Mu” (Compendium of Materia Medica), and “Yi Zong Jin Jian” (Golden Mirror of Medical Records), as the basic expansion library. Furthermore, formulas from classic medical cases are labeled as an extra expansion library.

#### 2.1.2 Data processing of core library and basic expansion library

This study, taking into account the characteristics of the selected ancient Chinese medical texts, first summarizes the entities, relationships, and attributes extracted from the medical data sources, and gradually abstracts and adds them to the data layer. Reading the literature and extracting knowledge entities involves fragmenting the literature, annotating the original text word by word, and extracting or summarizing it into TCM terms that contain only single information, which are knowledge entities. Knowledge entities must be terms or nouns that cannot be further decomposed. Building on previous construction of TCM knowledge graphs that only distinguished between clinical entities such as drugs and symptoms, this study’s entity extraction added categories including basic theories and medical cases, and further subdivided the entities of prescriptions, herbs, and formulas. The primary entities with a common theme in this literature were categorized, and the theme name was determined as the secondary entity; this principle was used to categorize and form higher-level entities such as tertiary and quaternary entities, ultimately classified as core entities. The research group, through expert demonstration, adopts a new entity extraction method: dividing the medical entities involved in the selected ancient texts into eight categories: disease names, dialectics, formula names, acupoints, external treatments, dynasties, physicians, and sources.

1. The first type of entity is the disease name. In this project, the disease name should include not only the name of the disease itself, but also the symptoms of the corresponding disease. As this project is only related to rheumatism, which is a modern medical name, rheumatism has complex naming in the Chinese medical system due to its location, etiology, syndrome, and disease characteristics. The standard name of the disease name should be the same as the name of Bi syndrome in the classification of TCM disease name terminology in GB/T 15657-202127 terminology category code.
2. The second type of entity is dialectics, and syndrome is a distinctive concept in traditional Chinese medicine. Syndrome is a diagnostic category used in clinical practice to summarize different stages and types of pathogenesis (including etiology, location, nature, and severity) in the disease process.
3. The third type of entity is the name of the formula, the name of the formula, and the name of the prescription. A prescription is a manifestation of therapeutic methods, which is a combination of several drugs based on compatibility principles and clinical experience.
4. The fourth kind of entity is acupoint, which is called acupoint in scientific name. It mainly refers to the special points on the meridian line of the human body. TCM can treat diseases by stimulating corresponding meridian points through acupuncture and moxibustion, massage, acupressure, and moxibustion.
5. The fifth type of entity is external treatment, which refers to the method of applying surgery on the surface of the body or using drugs or instruments to treat from outside the body outside of oral medication. As an important component of traditional Chinese medicine, external treatment is a concentrated manifestation of the characteristic treatment of traditional Chinese medicine.
6. The sixth type of entity refers to dynasties. As a discipline with a long history, traditional Chinese medicine requires special labeling of the time of literature. When reading and organizing information from ancient books, the uniqueness and limitations of the author’s era should be considered. In addition, due to the different scales of measurement in different dynasties, marking the era can also better help us understand the dosage of drugs.
7. The seventh type of entity is medical experts, and in the development of traditional Chinese medicine, there have been numerous brilliant figures, each with their own unique academic ideas; Due to constraints such as apprenticeship, medical environment, and historical conditions, it is inevitable that there may be some biases. While organizing the academic thoughts of medical experts, we should also annotate the theories of various medical experts to facilitate learners and researchers to objectively understand various ideas
8 .The eighth type of entity is the source, and in the process of establishing a knowledge graph, it is inevitable to fragment the knowledge, which may lead to unclear meanings. By annotating the source, it is convenient to quickly find the corresponding original text, facilitate learners and researchers to quickly locate the original text, and better understand the original meaning by reading through the context.

Through entity extraction, the knowledge of traditional Chinese medicine classics is fragmented and categorized, and the logical relationships among the fragmented knowledge are reconstructed. This method can determine the coverage of traditional Chinese medicine theoretical knowledge, efficiently collect domain terms, and is applicable to most fields of traditional Chinese medicine where categories are clear and entity relationships are distinct. Establishing relationships between entities involved creating relationships between entities after entity extraction. Building on previous research that simply described relationships between entities, this study further subdivided them into five major categories: subordinate relationships, causal relationships, formula-herb relationships, spatial relationships, and other relationships, with a further subdivision into 40 types of relationships. In computer visualization, these mainly manifest as one-way relationships, two-way relationships, reinforcing relationships, etc.

#### 2.1.3 Data processing of additional expansion database

Large Language Models (LLMs) have been widely applied in various fields, such as medical education and visual recognition (Liu et al., 2024a; Heston and Khun, 2023), but there is still a lack of relevant research in the field of automated construction of traditional Chinese medicine knowledge graphs. Due to the need for large language models to have a good understanding of ancient Chinese in the study of traditional Chinese medical texts, the progress of Chinese LLMs in recent years has provided a good technical foundation for constructing a knowledge graph of traditional Chinese medicine (Yi et al., 2024; Liu et al., 2024b; Xue and Zou, 2024). This study can explore the automation of the construction of a knowledge graph of traditional Chinese medicine through GPT 4.0.

##### 2.1.3.1 AI label data

Large language models can be effectively applied to knowledge extraction tasks. Since large language models are typically trained using a pre-training plus fine-tuning approach, they can fully utilize large-scale text data to model knowledge extraction tasks. We selected ChatGPT4.0 as the large language model for application, inputting relevant prompts to observe the output content. In specific experiments, the prompt consists of three parts:

1. Medical text to be identified.
2. Specific demand instructions.
3. Fine-Tuning Instructions.

This study creates the prompt for this research as GPTs instructions based on the text characteristics of traditional Chinese medicine ancient books and the table requirements of the Neo4j database. The prompt instructions are as follows: ① Utilize the NER function of NLP to extract key information about traditional Chinese medicine prescriptions from the text, identifying the following entity types: prescription name (e.g., “Guizhi Decoction”), source (e.g., extract “Sizhenjuewei, Volume 4″as the source), name of Chinese herb, dosage (e.g.,“1 liang”, “1 piece”), and disease condition (e.g.,“fengbi”). Establish the relationships between entities, the relationship between prescription name and source is set as “source relationship”, the relationship between prescription name and Chinese herb is set as “composition relationship”, the relationship between Chinese herb and dosage is set as “dosage relationship”, and the relationship between prescription name and main disease condition is set as “indications relationship”. ② Generate 2 knowledge graph tables that can be used for Neo4j. ③ Node table: The headers are “:id” (automatic coding), “:label” (entity type), “:title” (entity name); Relationship table: The headers are “:START_ID” (head entity, showing the id in the node table corresponding to the head entity), “:END_ID” (tail entity, showing the id in the node table corresponding to the tail entity), “:TYPE” (relationship). ④ The node table and relationship table are displayed in a table format.

Create My GPTs, enter the ChatGPT4.0 homepage, choose “Explore” to create a My GPTs, and enter the Configure interface to set the name, description, instructions, opening remarks, and other information which is the input for GPTs fine-tuning of the large language model.

Example 1: Construct a knowledge graph for the following text:

CinnamonTwig Decoction: Cinnamon (3 liang, peeled, pungent and hot in flavor) Peony (3 liang, bitter and sour in flavor, slightly cold in nature) Licorice (2 liang, roasted, sweet and neutral in flavor) Ginger (3 liang, sliced, pungent and warm in flavor) Jujube (12 pieces, broken, sweet and warm in flavor).

Return results:
Entity: CinnamonTwig Decoction
Relationship: Contains
Sub-entities: Cinnamon, Peony, Licorice, Ginger, Jujube
Entity: Cinnamon
Attribute: Dosage (3 liang), Processing method (peeled), Flavor (pungent and hot)
Entity: Peony
Attribute: Dosage (3 liang), Flavor (bitter and sour), Nature (slightly cold)
Entity: Licorice
Attribute: Dosage (2 liang), Processing method (roasted), Flavor (sweet and neutral)
Entity: Ginger
Attribute: Dosage (3 liang), Processing method (sliced), Flavor (pungent and warm)
Entity: Jujube
Attribute: Dosage (12 pieces), Processing method (broken), Flavor (sweet and warm)

ChatGPT4.0 has a certain degree of randomness, and fine-tuning is required during the labeling process. When the ChatGPT output is found to be irregular, instructions are given to standardize the names of entities, relationships, and categories according to the core library labeling method as the output format, allowing ChatGPT4.0 to output a second time to complete the fine-tuning of the large language model and continue with the labeling work.

##### 2.1.3.2 Manual review

**FIGURE 1:**
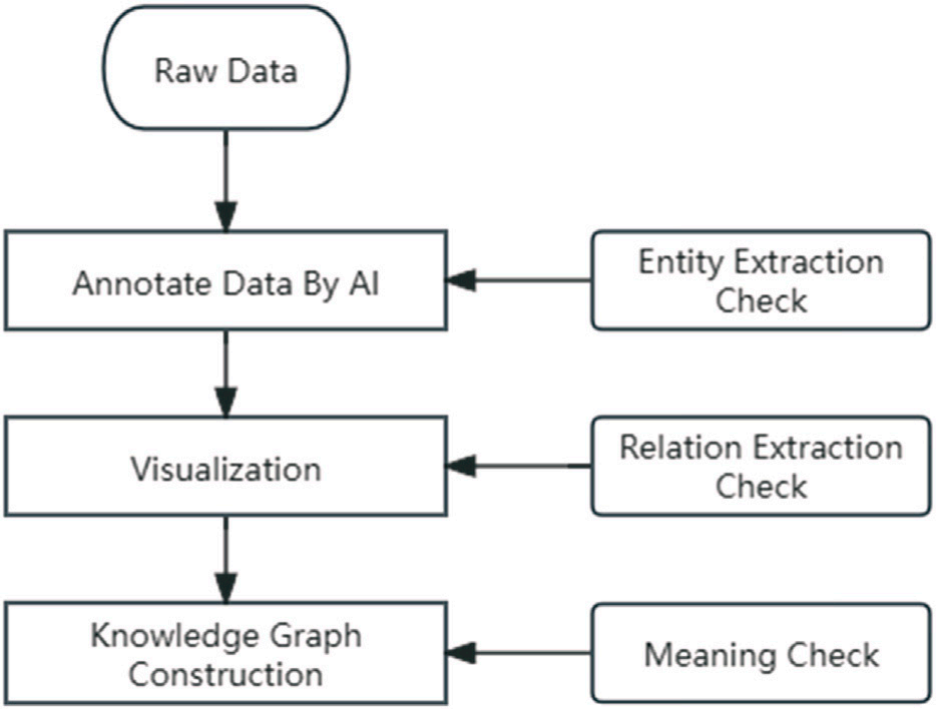
AI label data process.

Given the specificity of the medical field, it is imperative that medical knowledge graphs maintain the accuracy of professional knowledge. To better complete the task of medical knowledge graphing, this study designed a three-step data review system (Figure 1).

##### 2.1.3.3 First step review

Operators compare the AI labeling results with the manual labeling method to check if they meet the project team’s specified labeling standards and compare them with the original text to check for omissions.

##### 2.1.3.4 Second step review

Import the above results into the database, evaluate whether the relationships established are complete, and whether they comply with the labeling standards, supplementing or deleting incorrect relationships.

##### 2.1.3.5 Third step review

In the construction process of the TCMRD-KG knowledge graph, clinical staff and scientific researchers have engaged in deep and comprehensive collaboration. Clinical practitioners, with their rich front-line experience in diagnosis and treatment, have a keen and precise understanding of the symptoms, diagnostic processes, and treatment plans for various rheumatic diseases; while scientific researchers possess profound professional expertise in the design principles of knowledge graphs, data processing techniques, and information integration methods.

Both parties jointly undertake the rigorous evaluation of the final graph structure. They first examine the overall architecture of the knowledge graph from a macro perspective to see if it can intuitively and accurately reflect the clinical practice logic and research context of traditional Chinese medicine in the field of rheumatism. Then, they delve into the micro details of the graph, meticulously evaluating each entity, such as specific prescriptions, Chinese medicinal materials, and disease names, as well as the relationships constructed between them, such as the “indications” relationship between drugs and diseases, and the “composition” relationship between prescriptions and medicinal materials.

During the evaluation process, if any entities or relationships are found to be in obvious conflict with the professional knowledge of traditional Chinese medicine, a rigorous handling process is immediately initiated. For simple conflicts caused by data entry errors or misunderstandings, they are corrected on the spot. For controversial conflicts involving complex theoretical interpretations or the intersection of multiple knowledge systems, clinical and scientific researchers will organize special discussion meetings, inviting authoritative experts in the field to participate. Together, they analyze the root causes of the conflicts and, based on classical Chinese medical texts, modern clinical research results, and expert consensus, carefully make decisions on deletion or modification. Through this scientific, rigorous, and professional approach, they ensure that the knowledge graph aligns as much as possible with the actual needs of traditional Chinese medicine clinical practice and research, providing a solid and reliable foundation for subsequent applications.

### 2.2 Knowledge graph construction

**FIGURE 2:**
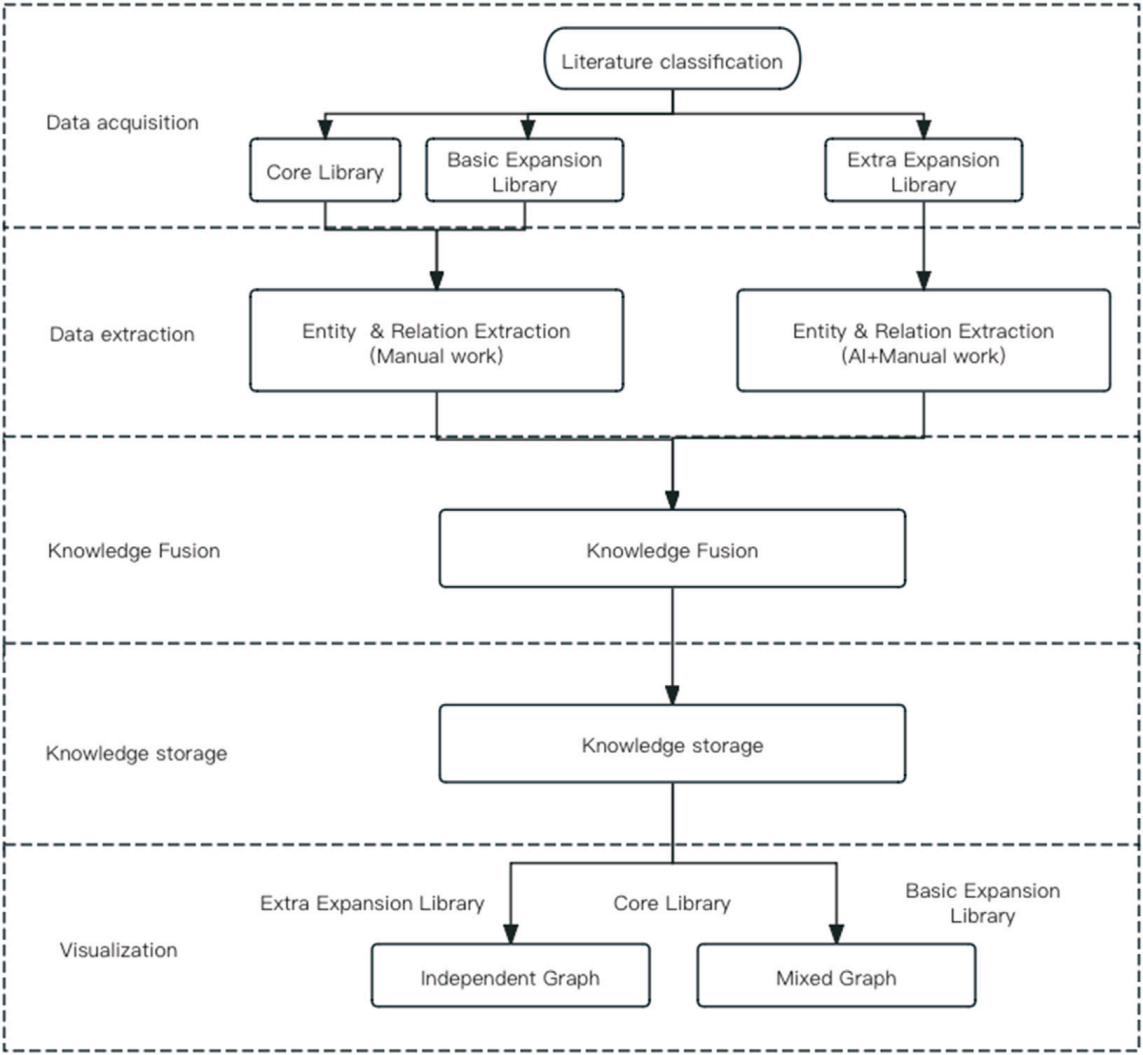
Knowledge graph construction process.

The traditional literature review has primarily focused on textual content research and has had low efficiency in clinical applications. The “Rheumatism Disease Syndrome Formula (Drug) Knowledge Management System,” a knowledge graph designed by the Institute of Basic Theory of Traditional Chinese Medicine, China Academy of Chinese Medical Sciences, aims to address this issue. This system collects, processes, and analyzes information related to rheumatism, including disease-syndrome-prescription-medicine, and presents all data through corresponding knowledge graph information. By systematically managing rheumatism-related knowledge information, it empowers relevant scientific researchers and frontline clinical staff (Figure 2).

#### 2.2.1 Data extraction

This study focuses on extracting entities and relationships from traditional Chinese medicine (TCM) texts related to rheumatism. The aim is to acquire knowledge about rheumatism from TCM ancient texts. A combination of manual labeling and AI entity-relationship recognition technology is used to extract structured data and construct several < entity-relationship-entity > data combinations. For the literature in the core library and basic expansion library, manual labeling is conducted according to Section 2.1. For the literature in the additional expansion library, further screening is performed to select prescription-related literature. The LLM (Large Language Model) model is used for knowledge labeling related to prescriptions, and the AI labeling results are manually reviewed.

#### 2.2.2 Knowledge fusion

This study employs a manual integration approach to configure and merge entities and their attributes based on business rules. Through multiple entity attribute comparisons and conflict detection, the study performs batch calculations to standardize and unify the names of extracted syndromes, symptoms, and other entities. This is done by referring to national standards for TCM clinical diagnosis and treatment terms (National Technical Supervision Bureau, 1997), common TCM clinical symptom terminology specifications (Jingbo and Li, 2015), and references such as “Chinese Pharmacology” (Zhong, 2016) and the “Chinese Pharmacopoeia” (National Pharmacopoeia Commission, 2015). The goal is to eliminate ambiguity, redundancy, and errors in concept representation. The calculated results are output to a fusion candidate set, which undergoes manual review before final merging.

#### 2.2.3 Data storage and retrieval

Neo4j is a graph database that is based on graph theory and implements a new type of NoSQL database. It has strong graph search capabilities and a certain degree of horizontal scalability. In the technical system of traditional Chinese medicine knowledge graph, Neo4j can serve as a storage repository for graph data, supporting various graph algorithms and applications. This study uses Neo4j graph database to store and retrieve the overall database; for the core library and other important knowledge graphs, it also provides two query languages: SPARQL and KGQL, for querying the graph knowledge, and supports the download of query results. This study are using of Neo4j architecture to display knowledge graphs and make modifications based on Neo4j.

## 3 Results

The core content of this study’s database is derived from the “Bi Syndrome (Rheumatism) Related Literature Compilation” compiled by the research group, which includes 378 ancient Chinese medical texts related to rheumatism. A total of 42,972 entities have been organized, and 41,253 relationships have been constructed, forming 1,100 knowledge graphs. The basic expansion library includes 34 classic ancient Chinese medical texts, with 149925 knowledge entities organized and 358,180 relationships constructed. These can be integrated and displayed as needed. The additional expansion library involves 10,468 prescriptions, with 58,079 related relationships constructed. It can be displayed independently (one prescription per graph) and can also be integrated and displayed with the core and basic expansion libraries.

This system provides external interfaces for data exchange, allowing it to connect with other databases or platforms to facilitate data exchange and queries. After knowledge construction, standardization classification, and knowledge metadata processing, all materials will be presented in a flattened manner through the recommendation and display subsystem. Knowledge relationship graphs are generated based on logical relationships such as basic theories, etiology and pathogenesis, syndromes, differentiation and treatment, treatment methods, diagnosis, acupuncture, acupoint therapy, medical cases, medical theories, prescriptions, and Chinese medicinal herbs.

In summary, a knowledge system based on ancient Chinese medical texts for the treatment of rheumatism has been established, integrating disease, syndrome, and prescription (medicine). The internal elements and relationships of TCM in the diagnosis and treatment of rheumatism are presented in the form of triples, which not only conforms to the rules of TCM syndrome differentiation and treatment but also facilitates further understanding of rheumatism knowledge. This provides a more intuitive and efficient method for the exploration and discovery of TCM knowledge related to rheumatism.

The additional expansion library was automatically annotated by AI and then manually reviewed to label the data. The text was filtered to only annotate entities related to prescriptions. This study selected the ChatGPT 4.0 model to complete the indexing task, which effectively achieved the extraction of content related to traditional Chinese medicine prescriptions and the necessary code for constructing the knowledge graph.

## 4 Discussion

### 4.1 This study is the first to construct a rheumatism-specific knowledge graph based on ancient Chinese medical texts

**FIGURE 3:**
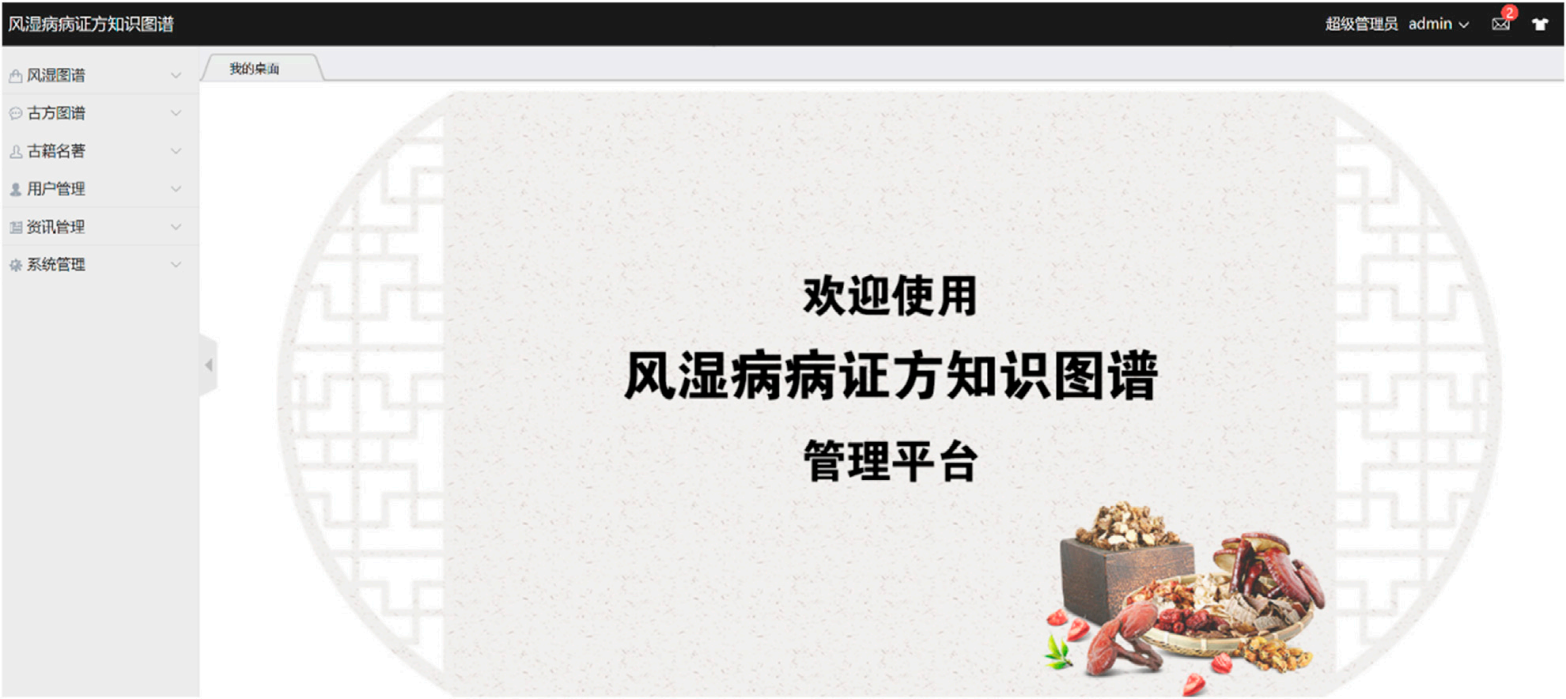
TCMRD-KG management platform display page.

**FIGURE 4:**
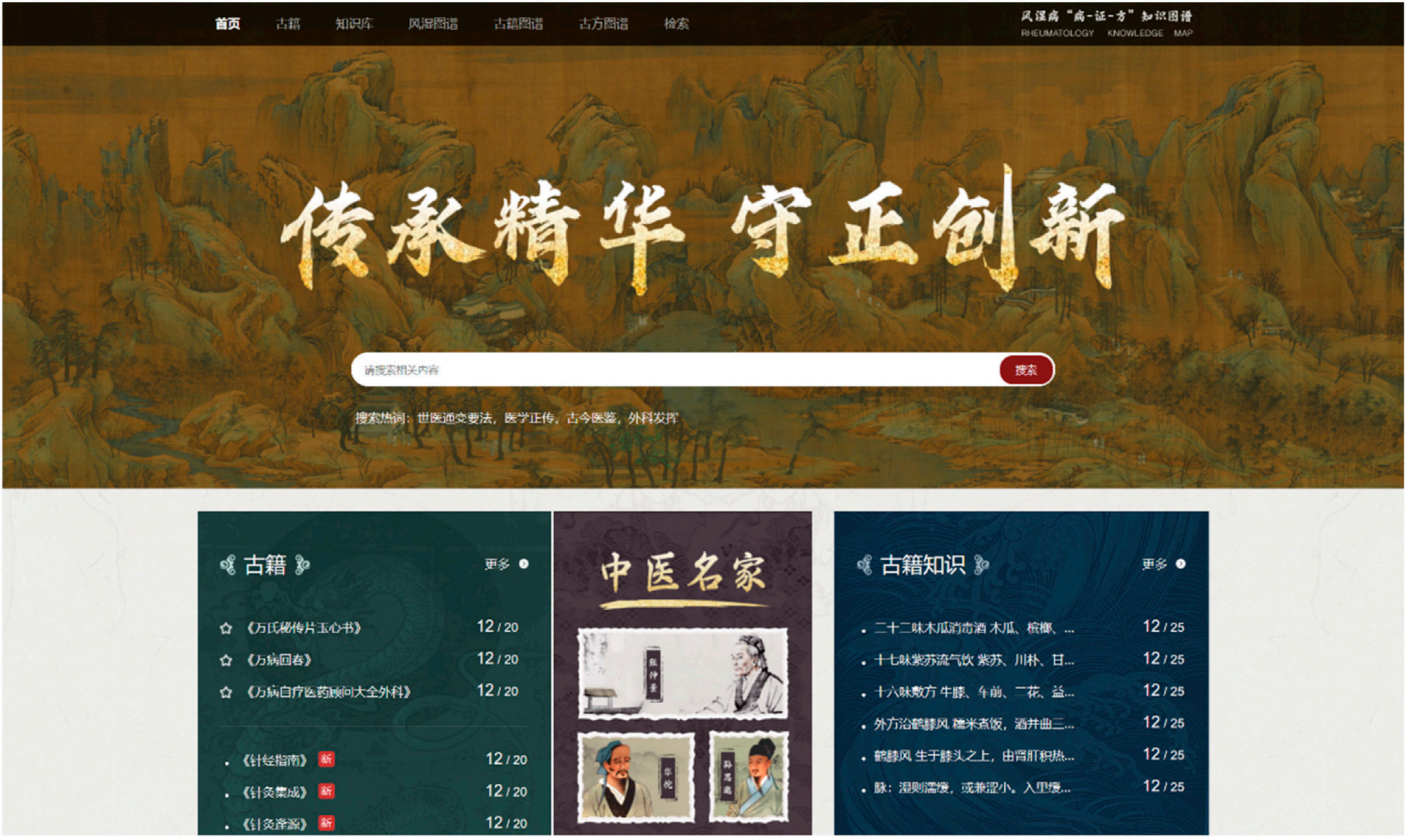
TCMRD-KG home page.

**FIGURE 5:**
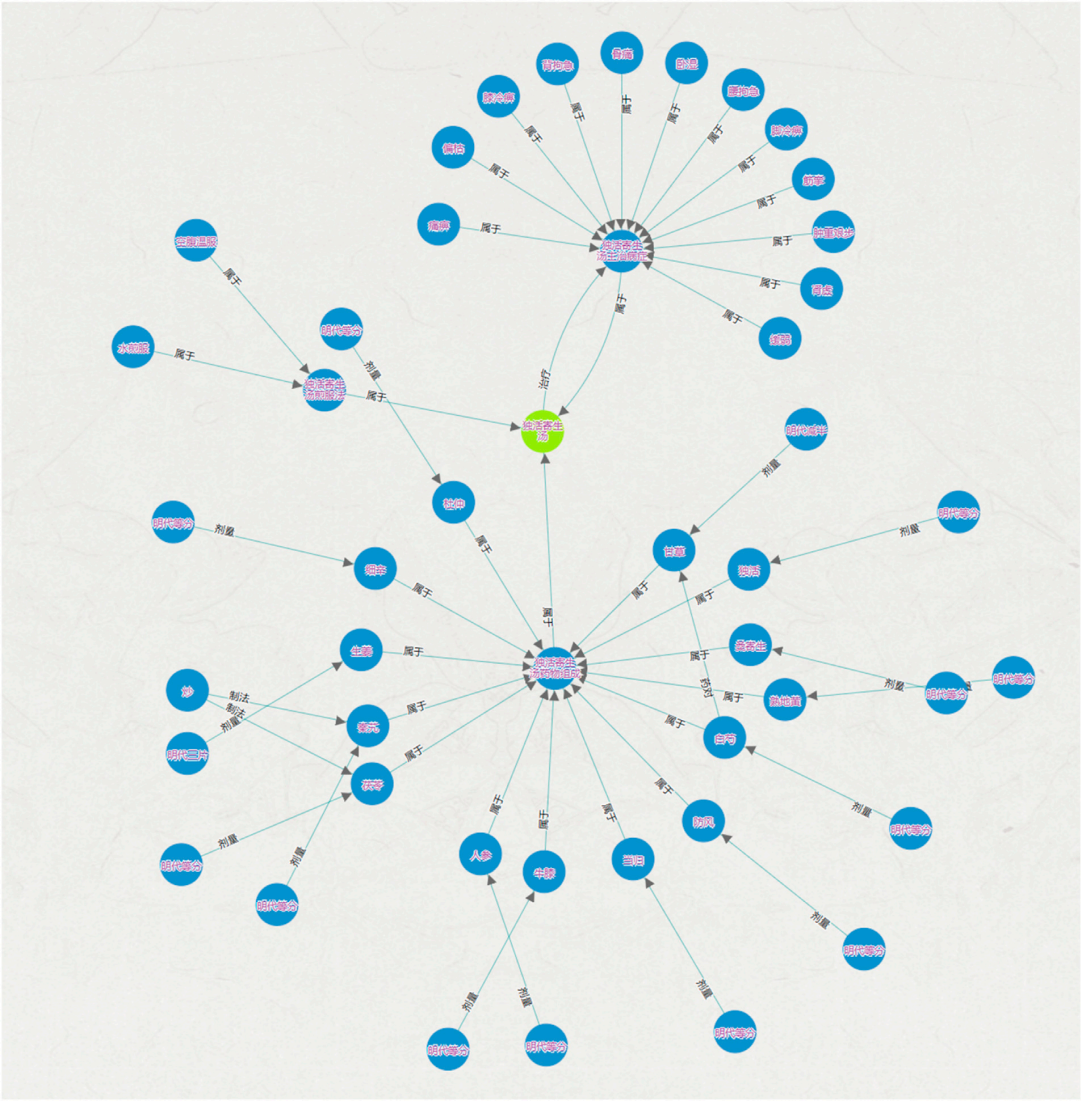
Schematic diagram of the core library map.

**FIGURE 6:**
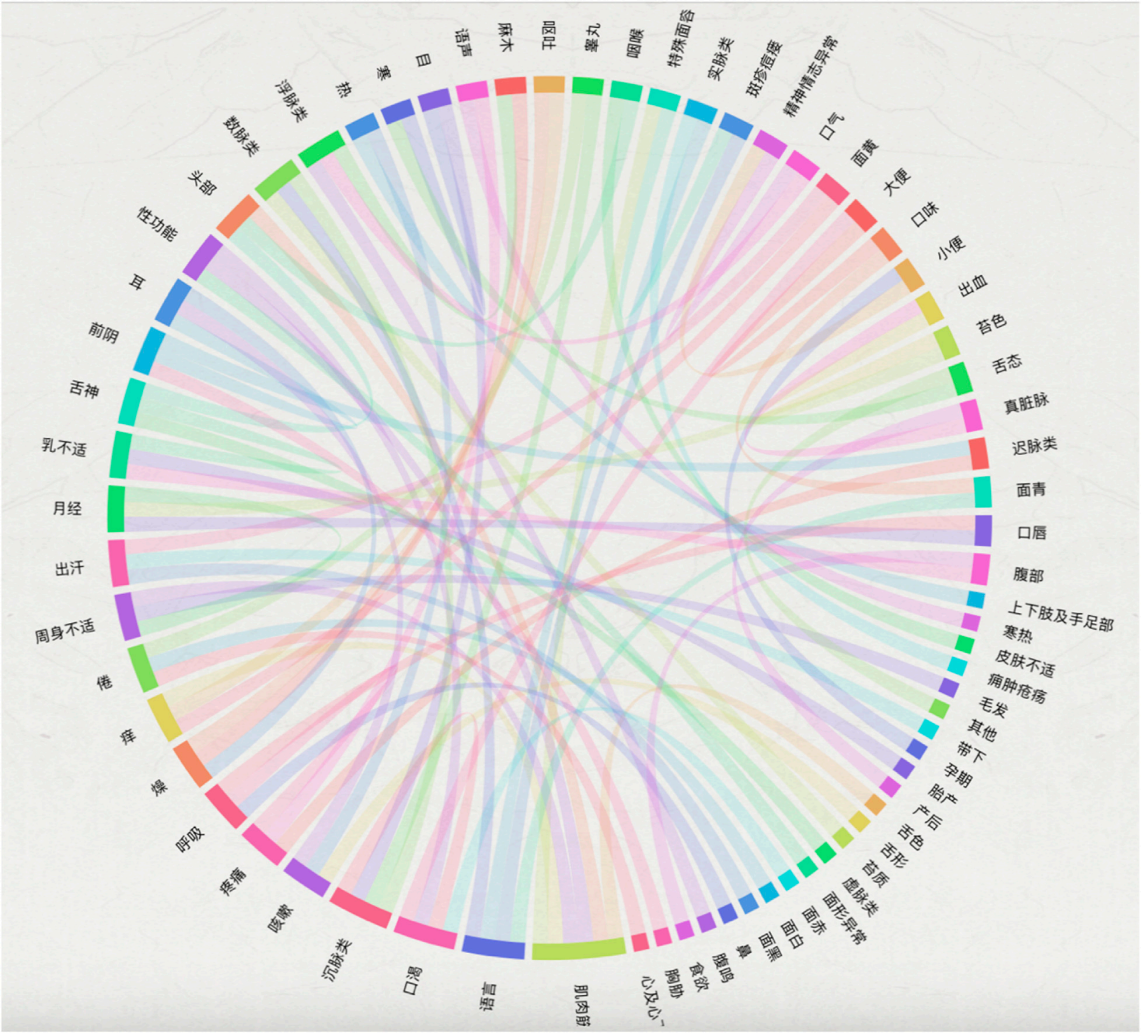
Entity-relationship diagram.

Ancient Chinese medical texts contain a vast amount of high-density TCM knowledge and information, which is of great significance to research and clinical practice. This study, focusing on the content related to rheumatism in ancient Chinese medical texts, complements it with classic TCM texts and related herbal prescriptions. It fully utilizes data mining, network feature analysis, and knowledge graph methods and technologies to build an ontology layer for TCM treatment of rheumatism based on the characteristics of the domain data. On this basis, semantic disambiguation and knowledge representation are carried out, and the structured data in the form of triples is stored and visually represented in the Neo4j graph database. This system is specifically designed to meet the “disease-syndrome-prescription (medicine)” retrieval needs of clinical professionals and related researchers in the field of rheumatism, constructing a complete and continuous knowledge spectrum diagram of rheumatism (Figures 3–6).

### 4.2 This study is the first to attempt a method of constructing a knowledge graph of ancient Chinese medical texts using mainstream large language models combined with manual review

Automated labeling is a popular research direction in the field of knowledge graphs. LLM can automatically extract knowledge points, entities, and relationships in the knowledge graph through the analysis and learning of a large amount of text data. It can interact with users through dialogue generation to optimize the accuracy and efficiency of relationship extraction. Since the advent of LLM such as ChatGPT, they have been widely used in information extraction. Thanks to the pre-training of LLM, it can perform simple entity and relationship recognition without the need for pre-training and parameter adjustment. However, for more complex custom rules, it requires a significant amount of time for fine-tuning and the results are poor. Therefore, this study uses large language models to replace the previous naming entity recognition (NER) techniques, directly performing entity and relationship identification related to prescriptions in the additional expansion library, and generating related code. To ensure the specialty and professionalism of the medical field, we have adopted a manual review mode for each AI identification result. This study has adopted a combination of manual labeling of the core library and AI labeling of the additional expansion library, significantly reducing the workload while ensuring the professionalism and effectiveness of the data.

### 4.3 This study proposes a refined method for labeling the knowledge graph of ancient Chinese medical texts

With a long history of the production of TCM literature, numerous texts, complex content and structure, and unclear propositions of various relationships, the complex logical relationships contained in TCM ancient texts are not fully expressed. In particular, the labeling of TCM ancient texts is challenging. Due to the development of TCM language, changes in clinical models, and other factors, there are significant differences between the names of entities and concepts in ancient and modern TCM knowledge. When extracting unstructured data, manual conversion and annotation of ancient and modern entity names are required. If we want to use computers to help obtain knowledge from ancient texts, we must establish a method of organizing knowledge deeply within the literature (Tao et al., 2022). Currently, the extraction of entities by computers is mainly based on the physical structure of the book itself, such as title, chapter, section, paragraph, sentence, and word, and then based on the logical structure of the text for semantic association, finally associating with knowledge elements or knowledge bodies. However, there is a lack of research on the internal relationships at the meta-concept level (Huang et al., 2021). This study further refines the labeling method through “entity-relationship-entity” or “entity-attribute-attribute value” labeling and can preserve as much as possible the objective knowledge contained in TCM. This can lay a solid data foundation for future clinical and basic research.

It should be noted that this study directly starts from the text content, extracts knowledge elements based on words or phrases, and then makes knowledge associations based on word relationships and text logic. This method to some extent makes up for the shortcomings of directly extracting entities, but it is easy to lose the objectivity expressed in the text content. Therefore, this labeling method requires a high level of professional quality from the labeler. This study innovatively introduces AI for automated labeling, but further research is still needed for complex texts and improving the accuracy of labeling.

## Data Availability

The original contributions presented in the study are included in the article/supplementary material, further inquiries can be directed to the corresponding authors.
